# Graft Suturing Method Affects on Graft Diameter in Hamstring-Based Anterior Cruciate Ligament Reconstruction

**DOI:** 10.7759/cureus.61054

**Published:** 2024-05-25

**Authors:** Takahiro Arakawa, Hisatada Hiraoka, So Kuribayashi, Shuji Okinaga

**Affiliations:** 1 Department of Orthopaedic Surgery, The University of Tokyo, Tokyo, JPN; 2 Department of Orthopaedic Surgery, Tokyo Teishin Hospital, Tokyo, JPN

**Keywords:** graft suturing, bone tunnel, double bundle, reconstruction, anterior cruciate ligament

## Abstract

Introduction: Various benefits of needleless suture loop techniques in anterior cruciate ligament reconstruction graft preparation have been discussed, yet their impact on graft diameter remains unexplored. We hypothesized that the suture loop technique would reduce the graft diameter compared to the conventional locking suture technique.

Methods: Fifty-seven patients whose grafts were made with the Krackow stitch (group K) and 54 patients with the suture loop (group SL) were analyzed retrospectively. (1) The distal (sutured side) diameter of each anteromedial bundle and posterolateral bundle was compared to the proximal (non-sutured side) diameter, and (2) the average of the proximal and distal graft diameters in each group was calculated.

Results: In group K, 78.9% of anteromedial bundles and 40.3% of posterolateral bundles exhibited a larger distal diameter than the proximal, while in group SL, 42.6% of anteromedial bundles and 3.7% of posterolateral bundles showed a larger distal diameter. In both bundles, there were significantly fewer grafts with larger distal diameters in group SL (p < 0.001). The mean distal diameter of anteromedial bundles was smaller in group SL (6.33 ± 0.43 mm vs. 6.07 ± 0.43 mm, p < 0.005). Consequently, the distal cross-sectional area of anteromedial bundles in group SL was 8% smaller than that in group K.

Conclusion: The use of the suture loop technique resulted in a significantly smaller distal diameter of the anteromedial bundle. This reduces the size of the tibial tunnel and may contribute to a reduction in potential damage to adjacent structures.

## Introduction

The principal aim of reconstructing the anterior cruciate ligament (ACL) is to restore knee stability and facilitate the successful resumption of sports and daily activities by patients [1-3]. Achieving successful ACL reconstruction is significantly influenced by accurately positioning the graft anatomically. Anatomical and biomechanical investigations have identified two primary bundles, namely the anteromedial bundle (AMB) and the posterolateral bundle (PLB) [4,5]. Consequently, a method for performing double-bundle ACL reconstruction was developed to replicate both bundles. Biomechanical studies have demonstrated that double-bundle ACL reconstruction provides superior rotational control compared to single-bundle reconstruction [6-8]. During the standard procedure for anatomic double-bundle ACL reconstruction using a hamstring autograft, surgeons typically employ either a locking or non-locking suture (e.g., Krackow stitch, baseball stitch, whipstitch, etc.) at both tendon ends. However, this approach may consume significant time and carry a risk of tendon injury during needle and suture passage through the graft [9,10]. Therefore, the advantages of various needleless suture loop techniques for hamstring tendon preparation have been the subject of debate [11-14]. Critical considerations include the elongation of the suture-tendon construct under cyclic loading, peak load to failure, and the time required for graft preparation. Nevertheless, there has been limited attention to graft diameters in these techniques. Because the suture loop constricts the tissue akin to a Chinese finger trap, there is a possibility of tissue compression leading to a reduction in graft diameter.

The purpose of this investigation was to examine potential disparities in distal graft diameters among different graft suturing techniques. We hypothesized that the distal graft diameter would be smaller in the suture loop technique compared to the conventional locking suture technique due to the volume of sutures used. If the graft diameter can be reduced, the bone tunnel will be smaller, which has the advantage of reducing tissue damage around the ACL footprint.

## Materials and methods

Patient characteristics

From December 2016 to December 2018, 124 patients underwent anatomic double-bundle ACL reconstruction using hamstring autografts at our institution, with a retrospective review of their medical records conducted. Thirteen cases were excluded due to the utilization of both the semitendinosus tendon (ST) and the gracilis tendon (GT), employing various graft configurations from GT (doubled, tripled, and quadrupled). Patients who underwent additional procedures, including partial meniscectomy, meniscal suture, medial meniscal posterior root repair, medial collateral ligament repair, posterolateral corner repair, and cartilage bone transplantation, were not excluded. Among the patients, 57 received grafts fashioned with the Krackow stitch (group K), while 54 underwent the suture loop technique (group SL) with the SpeedTrap Graft Preparation System (DePuy Synthes Mitek Sports Medicine, Raynham, MA, USA) (Video 1). SpeedTrap and Krackow reported no difference in the highest mean maximum load to failure and displacement during cyclic loading in graft fixation [15]. There were no statistically significant differences in age, sex, height, or body mass index between the two groups, as outlined in Table 1. Our study protocols received approval from the Tokyo Teishin Hospital institutional review board (number 1175), and patients were provided with study details and given the option to decline participation.

**Video 1:** Graft preparation method with Krackow stitch and suture loop method

**Table 1:** Patient characteristics Values are shown as n or mean ± standard deviation. Fisher’s exact test was used for gender, and the Student’s unpaired, two-tailed t-test was used for other items. Cm: centimeter; kg: kilogram; m: meter

	Group K (n=57)	Group SL (n=54)	P-value
Age (years)	31.1 ± 13.2	31.1 ± 12.3	1.000
Gender (Male/female)	29/28	28/26	1.000
Height (cm)	166.6 ± 10.3	166.7 ± 8.0	0.958
Body mass index (kg/m^2^)	23.9 ± 3.5	23.4 ± 3.7	0.445

Surgical procedure

The procurement of the semitendinosus tendon (ST) necessitated an anteromedial incision and an evaluation of its length. In cases where the ST did not meet the length criteria for double bundles, harvesting the gracilis tendon (GT) for the posterolateral bundle (PLB) became imperative, leading to exclusion from the study. When sufficient length was obtained from the ST, it was sectioned into two segments, with the distal portion designated for the anteromedial bundle (AMB) and the proximal portion for the PLB. Both bundles underwent identical preparation procedures. In group K, initiation of the Krackow stitch occurred 15 mm from the tendon end, incorporating at least three locking loops on each side using #2 FiberWire sutures (Arthrex, Naples, FL, USA). Conversely, in group SL, temporary sutures were utilized to secure both ends of the tendon. Subsequently, the tendon was looped over either an ACL TightRope RT (Arthrex) or an EndoButton CL (Smith & Nephew Endoscopy, Andover, MA, USA). Within group SL, the two sutured ends were unified using the SpeedTrap system, with the temporary sutures removed. The proximal and distal diameters of the AMB and PLB grafts were assessed at 0.5 mm intervals using a diameter measuring device, evaluating the diameters of cannulated reamers. The femoral and tibial bone tunnels were positioned at the center of each bundle's footprint. Femoral tunnel creation involved either an outside-in or trans-portal approach, followed by tibial tunnel formation from an outside-in direction. Subsequently, both grafts were passed through the tibial tunnels into the femoral tunnels. Tibial fixation was achieved using a Double Spike Plate (DSP; Meira Corp., Nagoya, Japan), applying an initial traction force of 30 N for each bundle. Graft tensioning was performed using a tensioning device, initially securing the PLB at 30° of knee flexion, followed by fixation of the AMB at 0° of knee flexion with the tibia in a neutral rotation.

Evaluation criteria

(1) A comparison was made between the diameter of the graft on the distal (sutured side) and that on the proximal (non-sutured side). (2) The mean diameters of the graft at both the proximal and distal ends were computed for each group.

Statistical analysis

Patient characteristics and study parameters underwent comparison utilizing the Student’s unpaired, two-tailed t-test or Fisher’s exact test. Histograms were generated to validate normality, and equivalency was confirmed via the F test. A p-value of <0.05 was selected as the level of significance. EZR statistical software, an open-source statistical program grounded in R and R Commander (Kanda, 2013), was employed for all data analysis.

## Results

**Figure 1:**
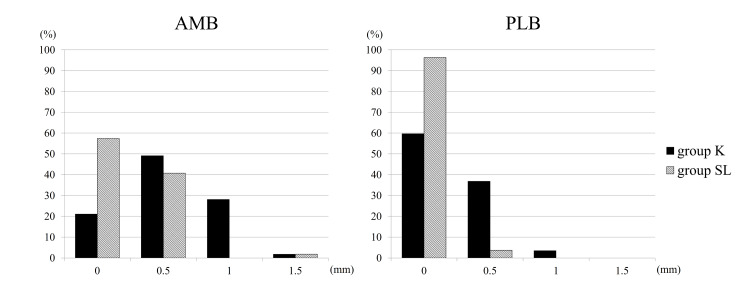
Differences between distal and proximal graft diameters The horizontal axis indicates how many millimeters larger the distal diameter is than the proximal diameter, and the vertical axis indicates the percentage of the group. AMB: anteromedial bundle; PLB: posterolateral bundle; mm: millimeter

**Figure 2:**
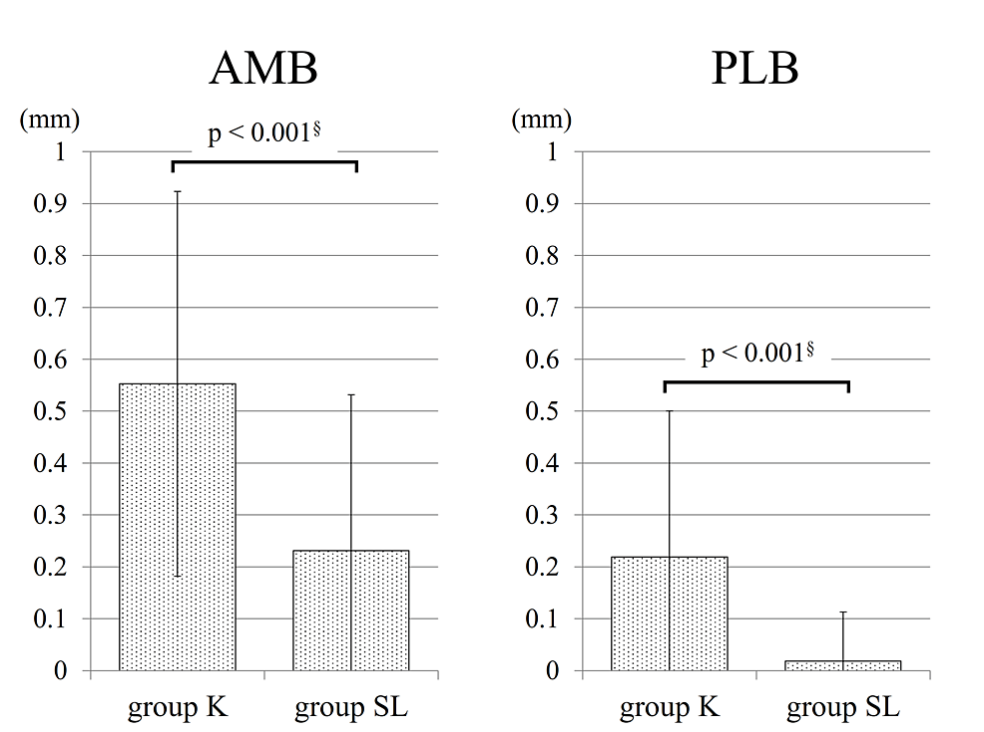
The average difference between proximal and distal graft diameters The horizontal axis indicates how many millimeters larger the distal diameter is than the proximal diameter. AMB: anteromedial bundle; PLB: posterolateral bundle; mm: millimeter

Within group K, 78.9% (45) of AMBs and 40.3% (23) of PLBs exhibited a greater distal graft diameter when compared to the proximal diameter. Conversely, in group SL, 42.6% (23) of AMBs and 3.7% (2) of PLBs showed a larger distal diameter. Group SL demonstrated a significantly lower occurrence of grafts with larger distal diameters in both bundles than group K (p < 0.001). The breakdown and average difference between proximal and distal graft diameters are depicted in Figures 1, 2.

**Figure 3:**
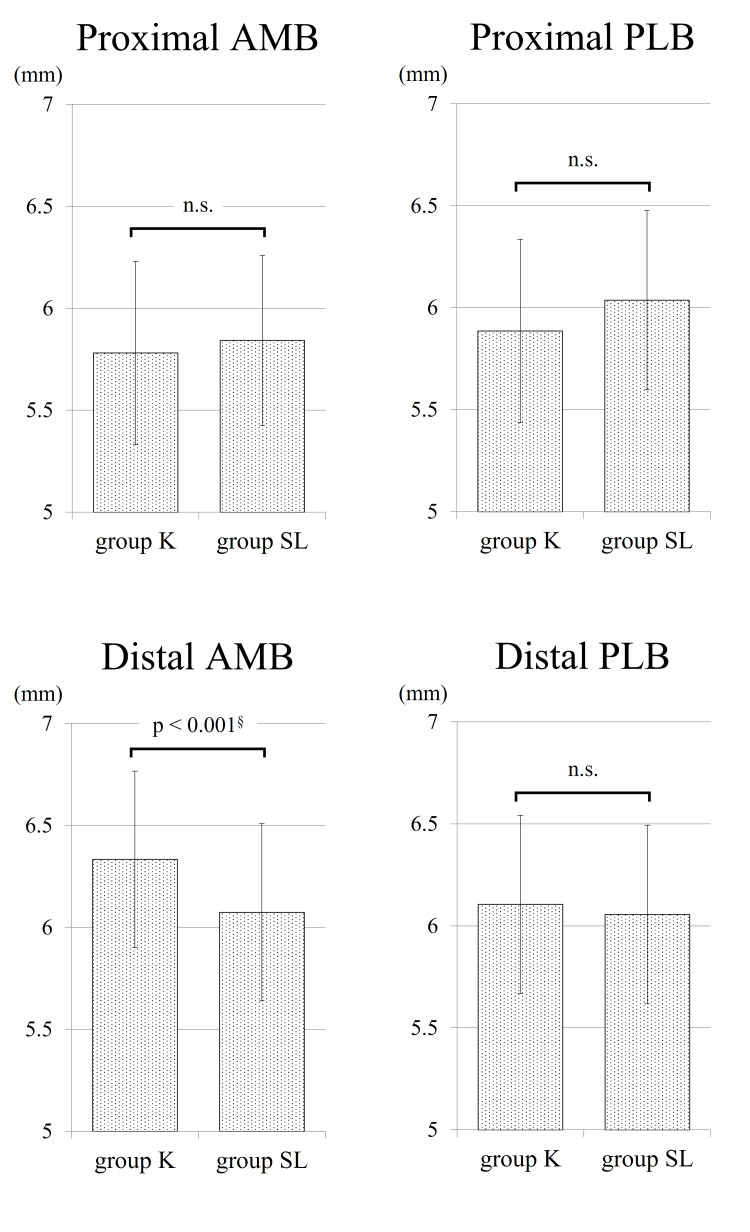
The average graft diameter The horizontal axis indicates the graft diameter. AMB: anteromedial bundle; PLB: posterolateral bundle; mm: millimeter; n.s.: not significant

**Figure 4:**
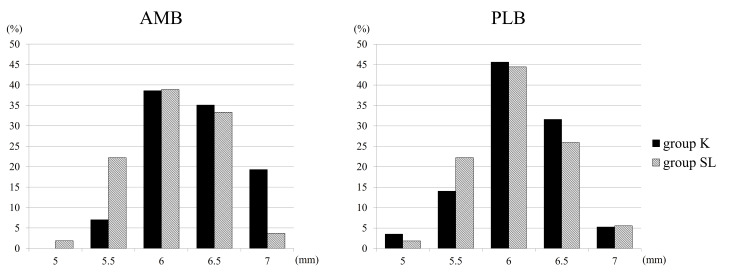
Comparing distal graft diameters between the two groups The horizontal axis indicates the distal diameter, and the vertical axis indicates the percentage of the group. AMB: anteromedial bundle; PLB: posterolateral bundle; mm: millimeter

The average proximal graft diameter for AMBs was 5.78 ± 0.45 mm in group K and 5.84 ± 0.42 mm in group SL (p = 0.459). Regarding AMBs, group K exhibited an average distal graft diameter of 6.33 ± 0.43 mm, while group SL showed 6.07 ± 0.43 mm (p < 0.001). Concerning PLBs, the average proximal graft diameter measured 5.89 ± 0.45 mm in group K and 6.04 ± 0.44 mm in group SL (p = 0.079). Similarly, the average distal graft diameter was 6.11 ± 0.44 mm in group K and 6.06 ± 0.44 mm in group SL (p = 0.554) (Figure 3). Distal graft diameters for each bundle are illustrated in Figure 4.

## Discussion

The most important finding of this study was that the average distal graft diameter of AMBs in group SL was significantly smaller than in group K (6.07 ± 0.43 mm vs. 6.33 ± 0.43 mm). Examination of the distal graft's cross-sectional area indicated an 8% reduction in AMBs in group SL compared to group K. This decrease in dimension might result in a smaller tibial tunnel, potentially decreasing the likelihood of harm to adjacent structures.

Extensive research has explored the anatomy surrounding the ACL and its attachment site. Initial studies proposed an oval or triangular shape for the tibial attachment of the ACL [16-19]. Siebold et al. introduced a "C"-shaped insertion along the medial tibial spine, with the LM anterior root situated at the center of the "C" [20]. LaPrade and colleagues further delineated that the LM anterior root lies deeply beneath the anatomical footprint of the tibial ACL attachment, exhibiting 63.2% overlap with the LM anterior root and 40.7% with the ACL footprint [19]. Additionally, they observed a 5-mm gap between the attachment centers of the LM anterior root and the ACL. In terms of the AMB, Zantop et al. noted its center positioned 2.7 mm posterior and 5.2 mm medial to the LM anterior horn center [16], while Ziegler et al. identified its location as 8.3 mm medial to the anterior-most fibers of the LM anterior root [17]. Due to the proximity of these structures, the LM anterior root is susceptible to injury during anatomic ACL reconstruction. Watson et al. were the first to report the potential risk of anterior meniscal root injury following ACL tibial tunnel reaming [21]. Recent studies have affirmed that a larger tibial tunnel diameter increases the risk of LM anterior root injury in both single-bundle and double-bundle ACL reconstructions [22,23]. In this examination, the cross-sectional area of distal grafts or tibial tunnel area, calculated from diameters, was found to be 8% smaller for AMBs in group SL compared to group K. Consequently, this graft preparation approach may mitigate the risk of LM anterior root injury.

Various researchers have explored different discoveries regarding techniques for graft suturing. Krackow et al. observed the failure of the Krackow stitch under cyclic loading conditions when subjected to loads exceeding 450 N [24]. In contrast, Wang et al. noted the higher peak tensile load due to failure and increased stiffness exhibited by native STs compared to those treated with interrupted sutures [25]. They proposed that interrupted sutures compromised the structural and mechanical integrity of the tendon due to the creation of multiple perforations during needle and suture passage. Su et al. introduced a novel suture loop technique called the modified finger trap (MFT), which eliminates the necessity for a needle. After undergoing 200 loading cycles, tendons sutured with the MFT technique demonstrated less elongation than those employing the Krackow and the locking Speed Whipstitch techniques. Furthermore, the MFT exhibited superior load-to-failure performance in comparison to other suturing methodologies [13]. Hong et al. evaluated the influence of suture throws in the suture-tendon construct for the MFT, the Krackow stitch, and the locking Speed Whipstitch. They discovered no significant variances in elongation following cyclic loading or load to failure among the scrutinized suture throw counts (3 vs. 5 vs. 7 throws) for the three suture types [26].

Our initial expectation was that there would be no significant differences in proximal graft diameters between the two groups, given the uniformity in proximal graft construction. Our data supported this hypothesis. In contrast to the AMB group, we did not observe any statistically significant variations in distal PLB diameter between the two groups. The exact reason for this outcome remains unclear, but it may be attributed to variances in donor sites. Since the distal ST served as the AMB, one end of the AMB included the periosteum, which likely exerted compressive forces. This factor may have influenced changes in graft diameter. Conversely, when examining the ratio of distal graft diameter to proximal graft diameter, the rate of expansion of the PLB in group SL (1.01 ± 0.03) was significantly lower than that in group K (1.08 ± 0.11) (p < 0.001).

This study has several limitations. First, graft diameters were measured at 0.5 mm intervals, a common practice that may not ensure precise diameter determination. Second, our surgical protocol required the diameters of tibial bone tunnels to match or exceed those of the femoral bone tunnels, as all grafts traversed from tibial to femoral tunnels. As a result, we did not assess whether distal graft diameters were smaller than proximal ones. This implies that some grafts may have had smaller distal diameters despite being considered equivalent to proximal diameters. Third, it was conducted retrospectively. This design can introduce certain biases, such as selection and recall bias, which might affect the validity and generalizability of the findings. Lastly, we did not examine the biomechanical properties of our graft suturing technique. While traditional suture loop methods typically involve placing the loop along each side of the tendon to create a doubled tendon graft, our technique with the suture loop differs in the arrangement. Therefore, further investigations are necessary to confirm its mechanical strength.

## Conclusions

The distal diameter of the AMB graft using the suture loop technique exhibited a notable reduction compared to the conventional locking suture technique. Consequently, the area of the AMB tibial bone tunnel in the suture loop technique was found to be 8% smaller than that in the conventional technique.
